# Optically Pumped Intensive Light Amplification from a Blue Oligomer

**DOI:** 10.3390/polym11101534

**Published:** 2019-09-20

**Authors:** Mamduh J. Aljaafreh, Saradh Prasad, Mohamad S. AlSalhi, Zeyad A. Alahmed, Muneerah M. Al-Mogren

**Affiliations:** 1 Department of Physics and Astronomy, College of Science, King Saud University, Riyadh 11451, Saudi Arabia; maljaafreh@ksu.edu.sa (M.J.A.); saradprasad@gmail.com (S.P.); zalahmed@ksu.edu.sa (Z.A.A.); 2 Research Chair on Laser Diagnosis of Cancers, Department of Physics and Astronomy, College of Science, King Saud University, Riyadh 11451, Saudi Arabia; 3 Chemistry Department, Faculty of Science, King Saud University, P.O. Box 2455, Riyadh 11451, Saudi Arabia; mmogren@ksu.edu.sa

**Keywords:** TD-DFT, conductive oligomer (CO) 1,4-Bis(9-ethyl-3-carbazo-vinylene)-9,9-dihexyl-fluorene (BECV-DHF), amplified spontaneous emission (ASE), time resolved spectroscopy (TRS)

## Abstract

We demonstrated the time-resolved dynamics of laser action from the conductive oligomer (CO) 1,4-Bis(9-ethyl-3-carbazo-vinylene)-9,9-dihexyl-fluorene (BECV-DHF). Absorption and fluorescence spectra were studied for BECV-DHF in different solvents under a wide range of concentrations. The Fourier-transform infrared spectroscopy (FTIR) spectrum was measured using simulation and experiments. The Ultraviolet-Visible (UV-VIS) spectra of the BECV-DHF were simulated in two different solutions. This CO formed a dimer and had two vibration bands in nonpolar solvents, partially dissolved in polar protic solvents, and created an H-type aggregate in polar aprotic solvents. BECV-DHF produced amplified spontaneous emission (ASE) at 464 nm in many solvents. The high efficiency of ASE is due to the waveguiding and self-assembly nature of the oligomer, which is very rare for optically pumped systems. However, BECV-DHF did not produce ASE in polar protic solvents. BECV-DHF produced ASE in both longitudinal and transverse pumping, and the full-width half maximum (FWHM) was 4 nm and 8 nm respectively for different solvents, such as toluene and acetone. The CO had a very low threshold pump energy (~0.5 mJ). The ASE efficiency was approximately 20%. The time-resolved spectroscopy (TRS) studies showed a temporal Gaussian-shaped ASE output from this CO. BECV-DHF shows remarkably high stability compare to the conjugated polymer (CP) PFO-co-pX.

## 1. Introduction

A material must possess various unique properties in order to be beneficial as a laser gain medium [1,2]. Foremost, the electronic structure for the material has to contain at least a two-level (or best a four-level) system such as a ruby crystal (conjugated polymers), so that the emission of these materials does not overlap with the absorption spectrum [2,3]. High luminescence and chromophore density are considered to be necessary conditions for laser gain materials; many conjugated materials stand out in this respect [4]. However, at high concentrations, the photoluminescence efficiencies drop for conjugated polymer laser chromophores, despite having the remarkable properties above. Another crucial property of any gain medium is that it must show a large cross-section stimulated emission, which is nominally in the range of σ = 10^−15^ cm^2^ for conjugated polymers [5,6]. This factor can affect laser action in terms of maximum gain, since the gain coefficient of the laser material (g = nσ) depends on σ and the density of electrons in the excited state (n).

The extraordinary photophysical properties of organic semiconductor materials have attracted a great deal of interest [2,7,8,9]. Conjugated materials have become highly desired materials for photonics devices, namely, light-emitting diodes [10,11], solar cells [12,13], organic field-effect transistors [14,15], and optical gain media, especially for lasers [16,17,18,19]. However, conjugated polymers (CPs) suffer from some disadvantages such as conformation irregularities, aggregation, low solvent solubility, uncontrollable polydispersity, and batch-to-batch irregularities. These disadvantages can be overcome in conductive oligomers (COs), in which significant advantages, such as a well-defined chemical structure, high self-aligning capabilities, chemical purity, and photophysical stability, are realized. However, the boundary between COs and CPs is not well defined, since the performance of the oligomers matches or exceeds the performance of the CP counterparts. Oligomers are a material that has a low molecular weight and contains a small number of repetitive units and a regular repeating structure (monodispersity and regioregular) [20,21,22]. Over the past decades, many detailed research papers have reported the amplified spontaneous emission (ASE) and the light amplification of oligomers [23,24,25,26]. ASE is considered to be a mirrorless laser, since most of the photons emitted are due to stimulated emission, and significant amplification is possible in a single pass [27]. Most of the organic semiconducting materials produce mirrorless lasers through a single trip of the laser light. When resonant feedback is combined with the CP or CO gain medium, it converts the ASE into an actual laser.

The ASE and spectral properties of the oligomer 9,9,9′,9′,9″,9″-hexakis(octyl)-2,7′,2′,7″-trifluorene (HOTF) under pulsed laser excitation in solution were studied [23]. The time-resolved spectroscopy of ASE from the conjugated oligomer 9,10-Bis[(9-ethyl-3-carbazoyl)-vinylenyl]-anthracene (BECVA) was reported only upon energy transfer [28]. Features of the unusually narrow emission from hexagonal 2,5-bis(4-biphenylylthiophene (BP1T) crystals were demonstrated under a line laser beam as the excitation source [29]. Lasing from single crystals of 2,5-bis(4′-cyanobiphenyl-4-yl)thiophene (BP1T-CN) was observed at room temperature. The results showed that the BP1T-CN crystal is an excellent gain medium, depending on high values of the group refractive index n_g_ (4.18–4.98) and high Q factor (910–1860) [30].

In this work, we report the spectral and laser properties of the CO 1,4-Bis(9-ethyl-3-carbazo-vinylene)-9,9-dihexyl-fluorene (BECV-DHF) in different solvents under pulsed laser excitation in both transverse and longitudinal excitation. The molecular structure optimization and UV-VIS calculations, Highest Occupied Molecular Orbital (HOMO) - Lowest Unoccupied Molecular Orbital (LUMO) energies, FTIR, absorption wavelengths, and oscillator strengths are theoretically analyzed and obtained by using the Time-dependent density functional theory/Becke, 3-parameter, Lee–Yang–Parr (TD-DFT/B3LYP) method with 6-31G (d) and 6-31G*(d,p) basis sets using Gaussian 09 [31]. The theoretical and experimental results presented are in good agreement with each other. Additionally, we show that under sufficient concentration and low pump energy, BECV-DHF can produce ASE at 464 nm in a large number of solvents. The ASE conversion efficiency was approximately 20%. A temporal Gaussian-shaped ASE output from this CO was noted through the time resolved spectroscopy (TRS) studies.

## 2. Materials and Methods

**Figure 1:**
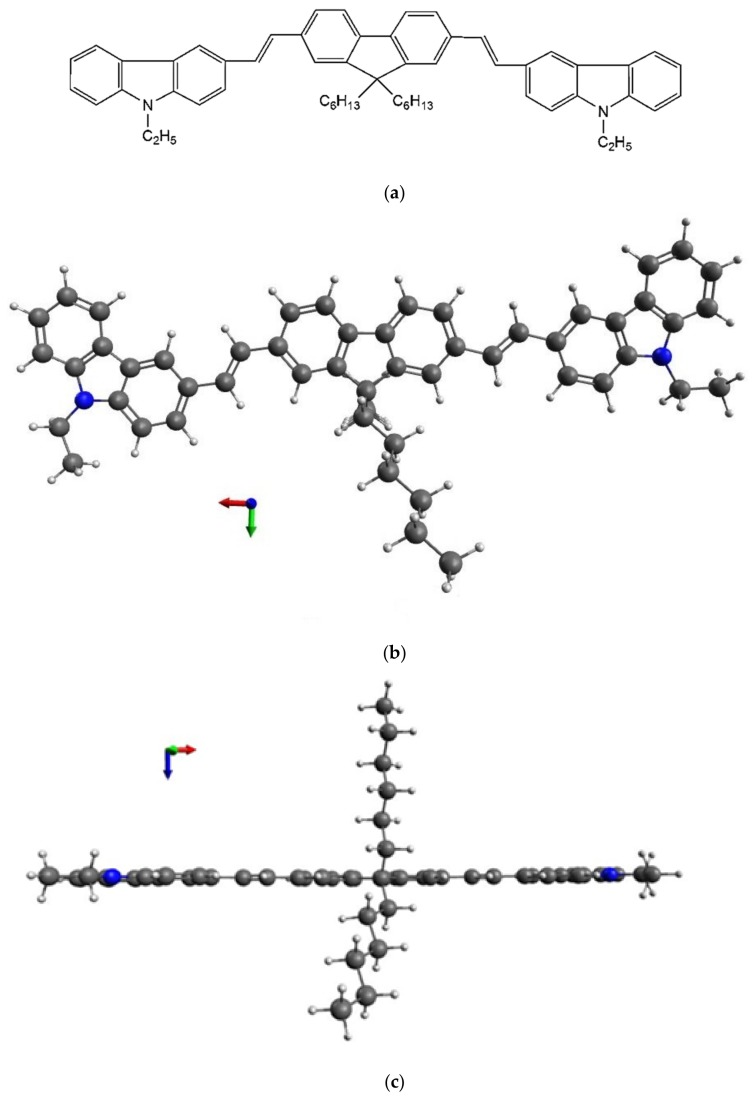
(**a**) The molecular structure of conductive oligomer (CO) 1,4-Bis(9-ethyl-3-carbazo-vinylene)-9,9-dihexyl-fluorene (BECV-DHF), (**b**) optimized geometry top view, and (**c**) bottom view using DFT calculation using universal force field (UFF) stepwise descent (Avogadro 1.1.1) and then using B3LYP/6-31G(d) (Gaussian 09).

Figure 1 shows the planar molecular structure the optimized geometric structure of the CO BECV-DHF, which is comparable with poly(o-phenylenediamine) oligomers [32] and Poly[(9,9-dioctylfluorenyl-2,7-diyl)-co-(4,4′-(N-(4-sec-butylphenyl)diphenylamine)] (TFB) polymer structures [33]. The molecule consists of a central core with 9,9-dihexyl-9*H*-fluorene with two 9-ethyl-9*H*-carbazoles on either side attached through (2*E*)-but-2-ene arms. It contains two long-chain hexane substituents on the fluorene core, which makes the material soluble in nonpolar solvents, and the carbazoles on either side have a high polarity that makes it soluble in polar solvents with excellent film-forming properties (as shown in Supplementary Figure S1a,b). This CO has many conformations, but Figure 1b,c shows the conformation with the lowest energy. Hence, the conformation in Figure 1b,c was chosen for simulation purposes. 

The CO had a molecular weight of 773.12 g mol^−1^, and CP poly[(9,9-dioctylfluorenyl-2,7-diyl)-co-(2,5-p-xylene)] (PFO-co-pX) had a molecular mass of 120,000 GPa (for molecular structure, please see [19]). They were imported from American Dye Source, Inc. (Quebec, QC, Canada). The spectroscopic grade solvents acetone, benzene, methanol (meth), dimethylformamide (DMF), and toluene were purchased from Sigma Aldrich. The TD-DFT calculations were done using Gaussian 09. For initial optimization, the open-source software Avogadro 1.1.1 was utilized. An advanced attenuated total reflectance (ATR) instrument with a range of 550–4000cm^−1^ was used to investigate the FTIR spectra of the CO BEVH-DHF. The ATR used for the FTIR experiment was a Perkin Elmer Spotlight 150i (Llantrisant, UK). A Perkin Elmer Lambda 950 spectrophotometer (Llantrisant, UK) was used to measure the absorption spectra across the 100–1100 nm range, and a spectrofluorometer (LS 55, from the same company) was utilized to measure the fluorescence spectra through a scan range of 200–1000 nm at room temperature. An energy meter was used to measure the input and output energy from the samples. 

The laser source for optical pumping was set to 5 ns pulse, 355 nm (frequency tripled Nd:YAG). The pump pulse from the Nd:YAG laser was focused by a 5 cm focal length quartz cylindrical lens (for transverse pumping) or spherical lens (for longitudinal pumping). The focused pulse with a shape line or cone was applied to excite the CO solutions. The CO solution produced laser-induced fluorescence (LIF) and ASE under suitable pump energies. The output light was fed to an optical fiber connected to an ultrafast camera (Princeton Instruments PI MAX 4 with an Acton spectrograph). The light signal was fed to a spectrograph with a linear array charge-coupled device (CCD) (Ocean Optics Spectroscopy, USB4000-XR1-ES, Ostfildern, Germany) to record the spectral features.

## 3. Results and Discussion

### 3.1. FTIR Investigation

**Figure 2:**
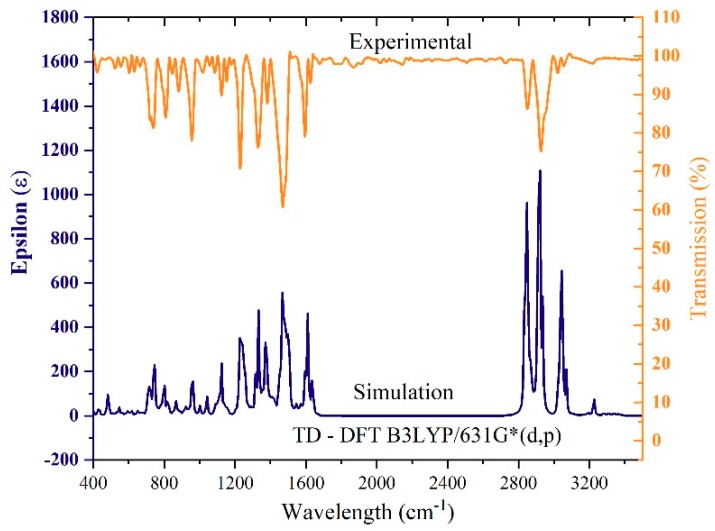
Simulated and experimental FTIR spectrum.

Figure 2 shows the FTIR spectrum of the CO BECV-DHF simulated (blue) using TD-DFT with the Becke three-parameter Lee–Yang–Parr B3LYP/6-31G*(d,p) basis set and experimental (orange) results measured using the FTIR instrument. The peak at approximately 3210–3220 cm^−1^ is due to stretching of the C-H bond in the aromatic rings (C10-H68, C9-H67) as presented in Figure S2. The peak at approximately 2916 cm^−1^ is due to stretching of the C-H bond at the tail of the atom (C57-C62, with H atoms). The peak at approximately 1230–1240 cm^−1^ is due to stretching of the C-C bond in the aromatic ring and due to bending of the C-H bond at the tail of the atom as shown in Figure S3. The peak at approximately 742 cm^−1^ represents the stretching of the C-C, C=H bonds in the ring, as demonstrated in Figure S4. The computational simulation and the experimental result were in agreement. For example, the longer wavelength peak at 2916 cm^−1^ in the experimental spectra is matched with 2929 cm^−1^ in the simulation, which indicates that a discrepancy between them is less than 1%. Note that a scaling factor was not used. The experimentally measured and simulated FTIR results showed a very close match, which confirms that the simulation method adopted for this CO is accurate.

### 3.2. Time-Dependent DFT Calculation of HOMO-LUMO

**Figure 3:**
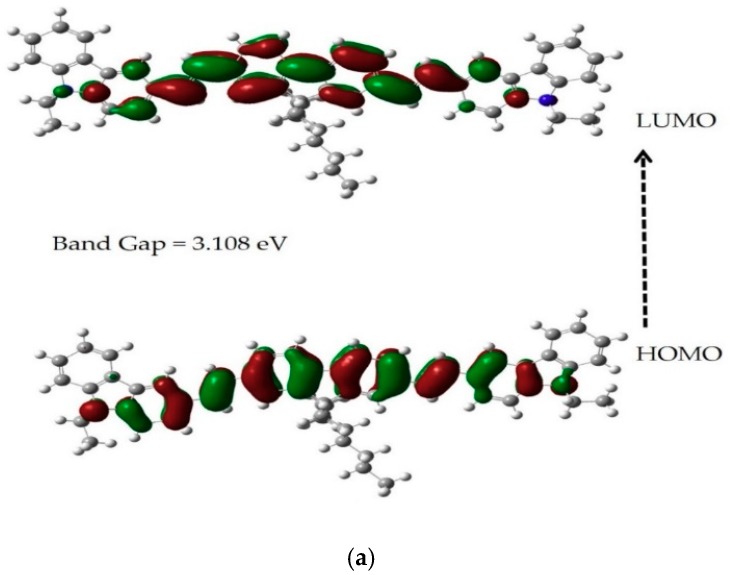

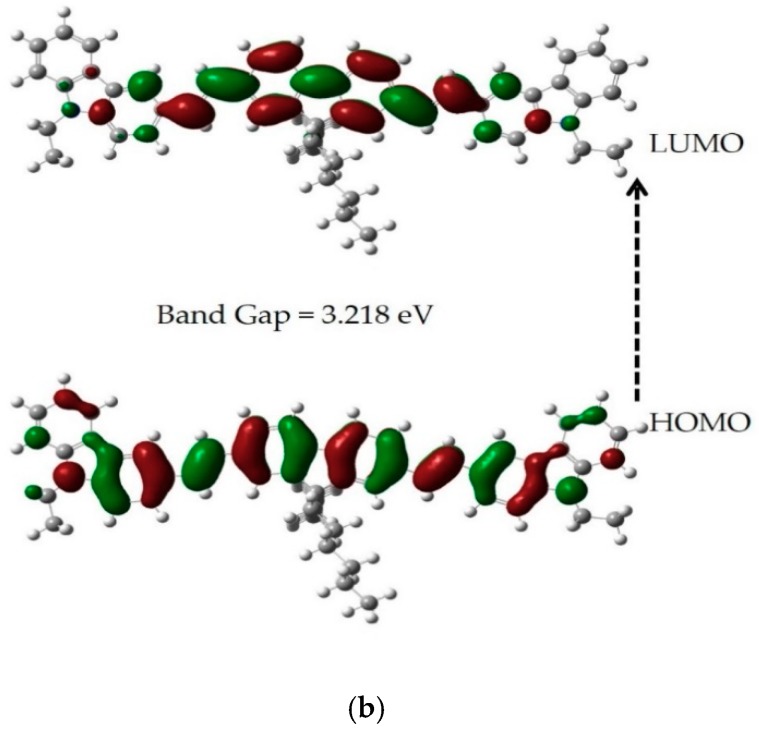
The HOMO-LUMO structure of CO BECV-DHF calculated using TD-DFT with B3LYP/6-31G* (d,p) basis set (**a**) toluene and (**b**) acetone cavitation.

The molecular orbitals provide great insight into electronic structures and are broadly used in the investigation of chemical reactions and physical properties. Figure 3a,b shows the HOMO-LUMO structure of the CO BECV-DHF calculated using TD-DFT with the B3LYP functional and 6-31G*(d.p) basis set. The geometry optimization and HOMO-LUMO structure were computed for CO under toluene and acetone cavitation. In toluene, as shown in Figure 3a, the HOMO-LUMO structure shows that the 9,9-dihexyl-9*H*-fluorene segment A contributed to the chromophore and that the 9-ethyl-9*H*-carbazole segments B have a little influence. In another study, we showed that a similar molecular structured CP PFO had an absorption peak at 360 nm, in which the 9,9-dioctyl-9*H*-fluorene segment was the entire contributor to the chromophore. Hence, the redshift of the absorption spectrum peak (398 nm) in BECV-DHF when compared to PFO is due to the contribution from Segment B. The long tail in segment A (i.e., 9,9-dihexyl) does not contribute to the fluorescence but could be the reason for the aggregation [34]. One can note that the LUMO contains a grouping of bonding orbitals for the C−C chemical bonds oriented along the long axis direction, while the HOMO contains a combination of bonding orbitals for C−C chemical bonds oriented along the short axis, which can be seen in other conjugated materials [35].

**Figure 4:**
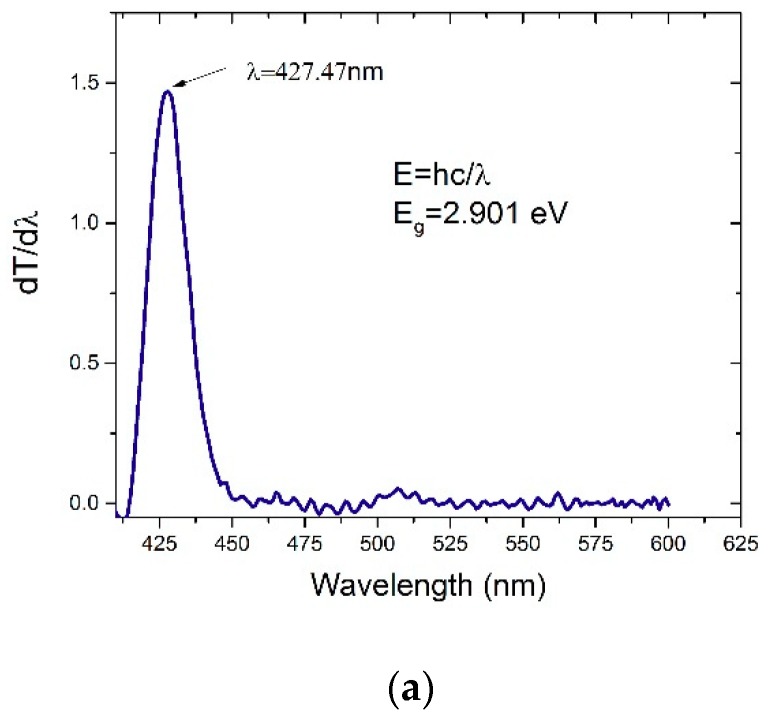

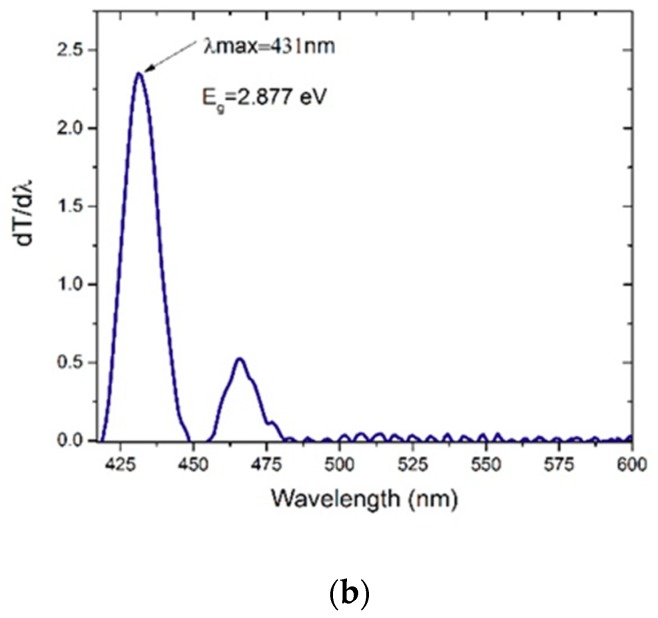
The variation of dT/dλ vs. wavelength CO in (**a**) acetone and (**b**) toluene.

The HOMO-LUMO structure of the CO BECV-DHF for acetone cavitation was calculated using TD-DFT with the B3LYP/6-31G* (d,p)basis set as presented in Figure 3b. The contour of the HOMO is same in acetone when compared to toluene, except that segment B (9-ethyl-9*H*-carbazole) has more electron density. The computed HOMO-LUMO gap is consistent with the experimental results of CO in the two solvents, as in Figure 4a,b. The energy gap (E_g_) of the CO was found from the derivative of transmission and calculated using the relation E_g_ = hc/λ_max_. The derivative of transmission over the photon wavelength (dT/dλ) peak corresponded to the bandgap of the CO. The difference in the simulated and observed bandgap is because the experimental bandgap is obtained for a group of CO molecules in a large volume of solution, but computation is based on a single molecule with solutions cavitation. However, the difference is in an acceptable range. The results reveal that the λ_max_ for CO in toluene and acetone (for the same concentration of 1.235 µM) was 431 and 427.47 nm, respectively. The band gaps corresponding to these wavelengths were 2.877 eV and 2.901 eV, respectively, as shown in Figure 4a,b, with a discrepancy of 7.4% and 9.85% when compared to the stimulation. It is also possible to find the CO optical energy gap by crossing the absorption and fluorescence spectra for 1.235 µM in the same solvents with Eg = 2.877 eV in toluene and Eg = 2.897 eV in acetone, as presented in Figures S5 and S6.

### 3.3. Time-Dependent DFT of CO’s Energies

**Figure 5:**
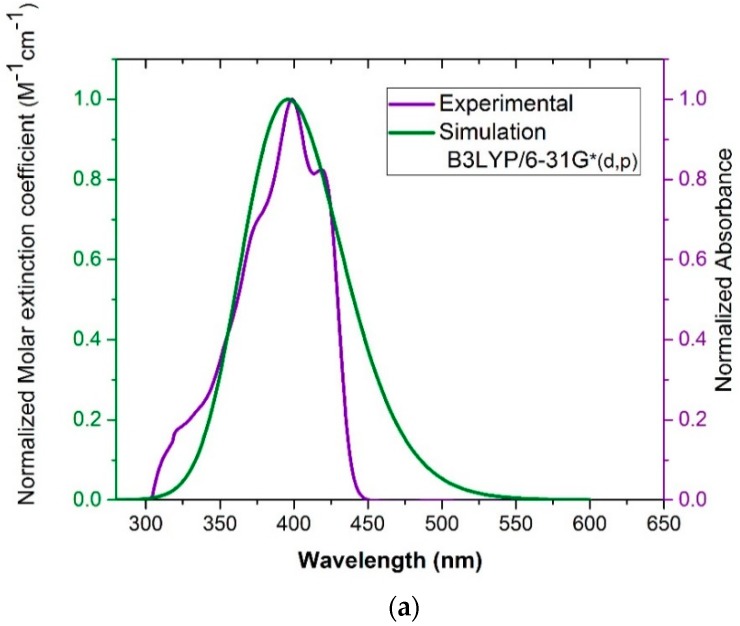

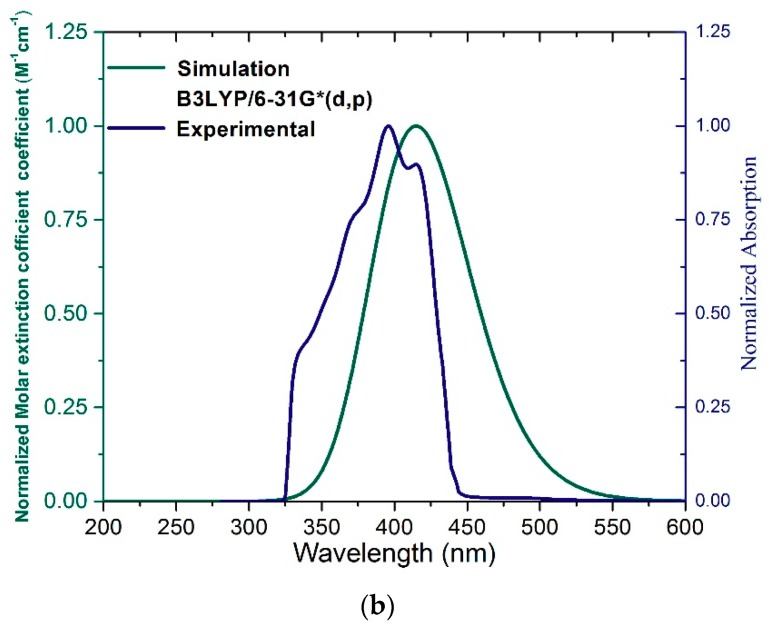
Comparison of the simulation and experimental absorption spectra in (**a**) toluene and (**b**) acetone.

Figure 5a shows the simulated and experimental spectral profile of BECV-DHF in toluene. The simulation was done using TD-DFT with the B3LYP/6-31G* (d,p) (green line) basis sets and toluene cavitation. The experimental spectrum was measured at a low concentration (7.58 µM) of CO in toluene. The oscillator strength and absorbance serve the same purpose because the oscillator strength is utilized as a scale of the relative strength of the electronic transitions within the molecular and atomic systems. The simulation based on B3LYP/6-31G* (d,p) gave only one peak at 389 nm, with an oscillator strength (f) of 2.1718. The theoretically simulated λ_max_ at 395 nm showed a strong correlation with the experimental λ_max_ at 398 with a 3 nm difference. The full-width half-maximum (FWHM) of the experimental results (~67 nm) was narrower than that of the simulated spectrum (~79 nm). Figure 4b shows the experimental absorption spectrum (blue line) of a low concentration (7.58 µM) of CO in acetone and the simulated absorption spectrum (green line) of CO in an acetone cavity using B3LYP/6-31G* (d,p) as the basis set. The simulated spectrum contained one peak at 415 nm with an oscillator strength (f) of 2.526, which is the closest compared to the experimental absorption band at 415 nm and has a variation of 20 with the experimental λ_max_ at 395 nm. The FWHM of the simulated spectrum (~81 nm) was comparable to the experimental results (~80 nm). The simulation shows that the oscillator strength of CO in nonpolar and polar solvents was high and almost equal.

### 3.4. Experimental Absorption Spectra in Solution

**Figure 6:**
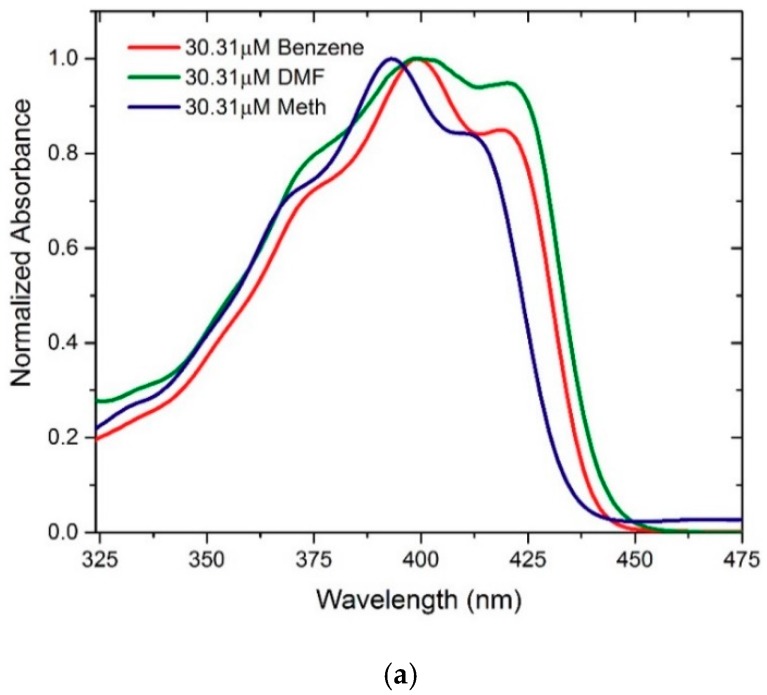

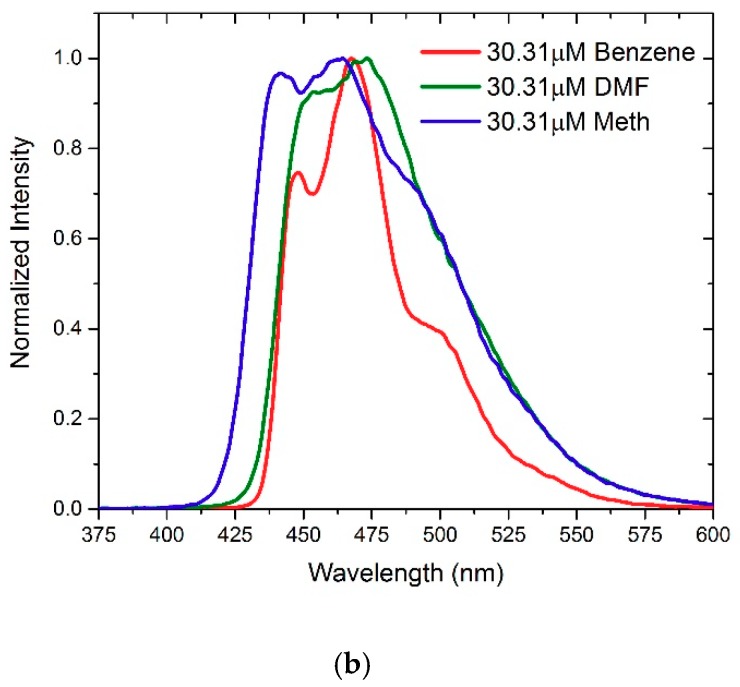
(**a**) Absorption and (**b**) fluorescence spectra of BECV-DHF in three different solvents at a concentration of 30.31 μM.

Figure 6a shows the absorption spectra for three different solvents, where the blue line is the spectral profile of CO in methanol (a polar protic solvent), with a peak at 393 nm and two humps at approximately 371 and 409 nm on either side of the peak, and the FWHM was 66.5 nm. The green line is the spectral profile of CO in DMF (a polar aprotic solvent), with a peak at 400 nm and humps at approximately 374 and 421 nm, and the FWHM was 78.6 nm. In addition, the red line is the spectral profile of CO in benzene (a nonpolar solvent), with a peak at 400 nm and humps at approximately 373 and 421 nm, and the FWHM was 70 nm. The absorbance spectra of the polar aprotic and nonpolar solvents showed a very similar profile with well distinguishable peak and humps, but for the polar protic solvent spectra are slightly shifted in relative to other solutions and the profile was narrow as well as the features were distinctive. The hump at 373 nm is due to vibronic band S0-S1 of monomer, the peak at 400 is due to vibronic band S0-S2 of monomer, and the hump at 421nm is attributed to the dimer. Section 3.5 describes these two humps and the peak. 

The fluorescence spectra for three different solvents specified is given in Figure 6b. The interval between the peaks in the fluorescence spectra in the three solvents is analogous to the three features in the absorption spectra. The fluorescence profile of CO in methanol had two peaks at 441 and 463 nm along with a hump in the longer wavelength region at approximately 486 nm; the fluorescence FWHM was 77 nm. The spectra of CO in DMF had a hump at approximately 455 nm and a peak at 473 nm with an FWHM of 68 nm. The profile of CO in benzene (a nonpolar solvent) had a peak at 467 nm and humps at 448 and 497 nm; the fluorescence FWHM was 44 nm. The fluorescence spectra of polar protic and nonpolar solvents showed a very similar profile with well distinguishable peak and humps, but for polar aprotic solvents, the profile was broad, the features were not distinctive, and the peak at approximately 430 nm vanished. It is known that primary vibronic transition (V1) is forbidden when a material forms H-aggregates; hence, the absence of the S0-S1 transition attributes to the formation of H-aggregates in the polar aprotic solvent [36].

### 3.5. Absorption and Fluorescence Spectra of CO in Toluene

**Figure 7:**
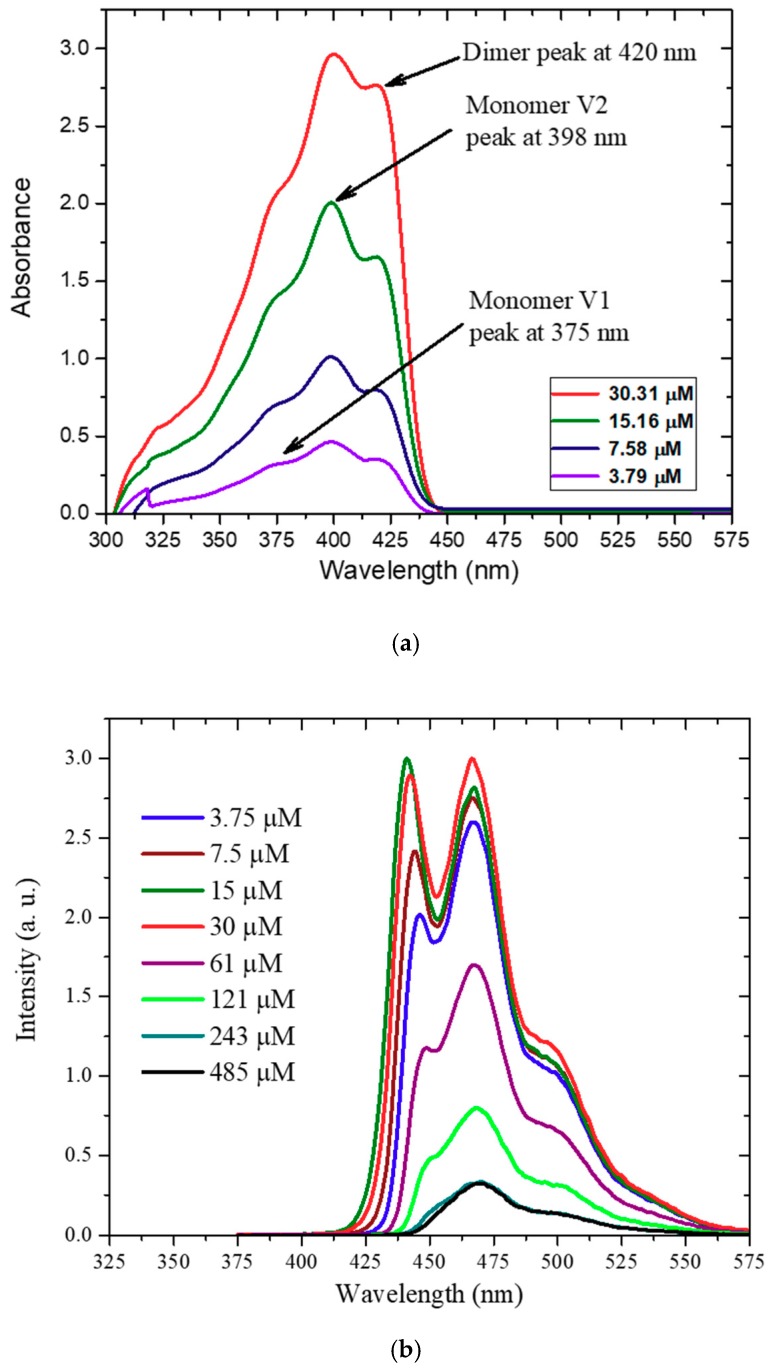
(**a**) Absorption spectra of BECV-DHF in toluene for different concentrations. (**b**) Fluorescence spectra of BECV-DHF in toluene for different concentrations.

The range of concentration used was 485–3.79 µM, a 128-fold dilution. The absorption spectrum for higher concentrations (485–60.63 µM) was saturated. For concentrations less than 30.31 µM, the absorption spectra showed three different features as in Figure 7a: a shoulder at a shorter wavelength of approximately 375 nm, a peak at 398 nm, and another shoulder at a longer wavelength of approximately 420 nm. These features are attributed to the vibration band S0-S1 (V1) of the monomer and the vibration band S0-S2 (V2) of the monomer, and the hump at 420nm is attributed to the dimer. The ratio vibration peaks of the monomer did not change (i.e., $R_{\frac{375\;nm}{398\;nm}}=0.69$) for all concentrations, hence it should be the vibrational features of the monomer. However, the ratio between the monomer peak at 398 nm and the dimer at 420 nm ($R_{\frac{420\;nm}{398\;nm}}$) is decreased from 93% to 75% for the concentrations shown above. This steady decline of the peak absorption with respect to concentration is evidence for the presences of the “dimer” [37]. This aggregation could be explained in terms of β-phase formation due to the side legs (C_8_H_17_) [38]. 

Figure 7b presents the fluorescence spectra of CO, which display the mirror image to the absorption spectra. It shows two well-defined peaks around 441 and 466 nm, corresponds to the vibrational features V1 and V2 explained in the absorption spectra in Figure 7a. The fluorescence showed a hump at approximately 496 nm, attributed to the dimer. The fluorescence quantum yield of the CO BECV-DHF was calculated in both solutions (toluene and acetone at a concentration of 1.15 µM), and the results were 0.96 and 0.9, respectively. The spectral profile for CO in acetone is shown in Figure S7 and points to the formation of H-aggregates.

### 3.6. ASE (Mirrorless Laser) from CO BECV-DHF in Toluene and Acetone

**Figure 8:**
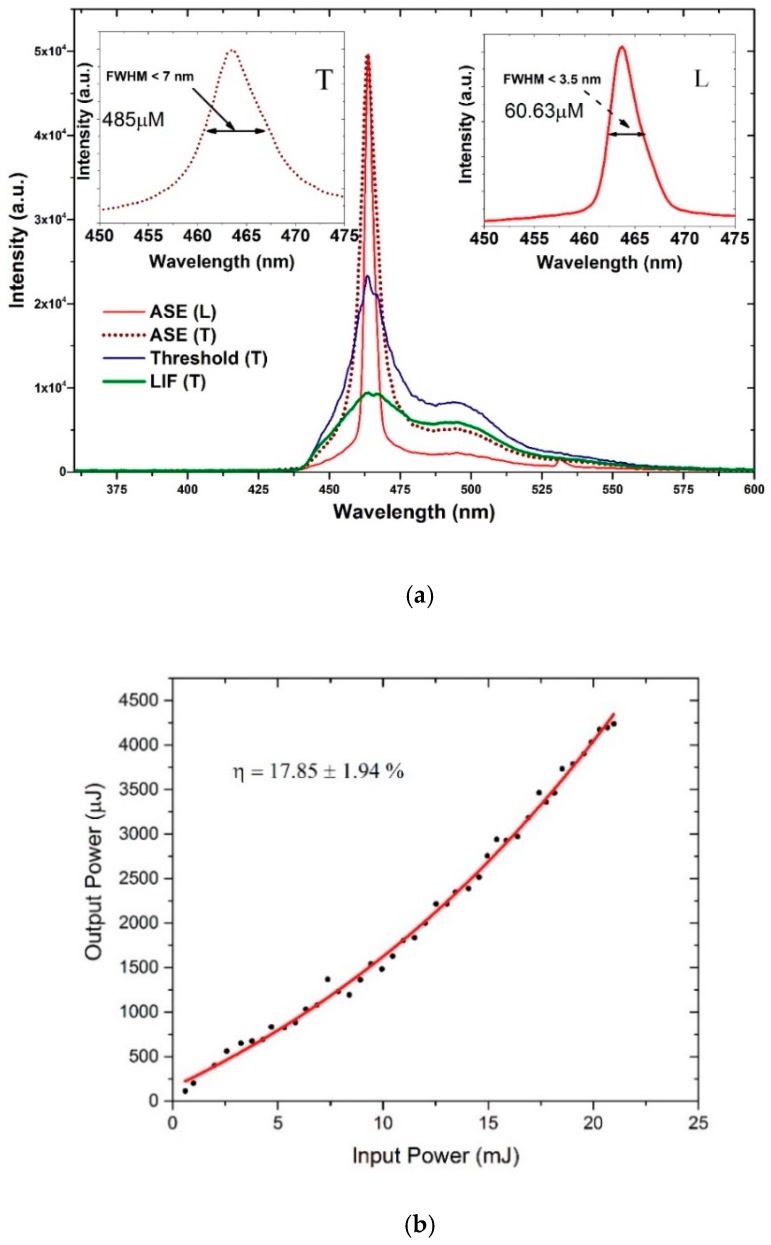

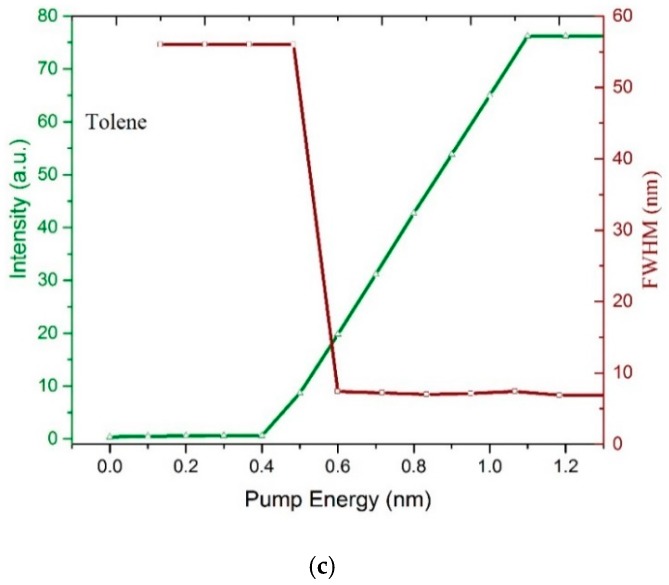
(**a**) ASE, threshold and laser-induced fluorescence (LIF) spectra of CO in toluene, (**b**) efficiency of CO ASE in toluene with input energy (mJ) versus output energy (μJ), and (**c**) relationship between pump energy, intensity, and full-width half maximum (FWHM) of ASE in toluene.

The laser spectra for the CO in many solvents were obtained. The importance of this oligomer arises from a rare property that it can produce ASE from both polar and nonpolar solvents. Hence, we describe the photophysical properties of this oligomer in two different categories of the solvents acetone (polar, aprotic) and toluene (nonpolar). The CO kept in toluene at a concentration of 485 µm was transverse-pumped with an energy of 0.3 mJ to obtain laser-induced fluorescence (LIF) with an FWHM of 54 nm. When the pump energy (PE) was increased to 0.5 mJ, a spectral narrowing of 18 nm occurred. A further increase in PE (0.8 mJ) produced an efficient ASE peak at 464 nm with an FWHM of 7.4 nm, and the divergence of the narrow beam was 5 milliradians (mr), as shown in Figure 8a. Hence, this CO has a four-level laser system permitting a low-threshold process. 

In the case of longitudinal pumping, the concentration of the solution was six times lower than that of the transverse (60.63 µM) to allow deeper penetration of the pump beam and to avoid strong reabsorption due to attenuation of the active medium. The minimum pump energy for laser action was 1.6 mJ; the ASE had an FWHM of 6 nm. When the pump energy increased to 2.5 mJ, a very narrow ASE spectrum was produced with an FWHM of 3.5 nm, as presented in Figure 8a. The low threshold is could be due to efficient waveguiding and small feedback from the faces of the cuvette. The narrow emission of the CO in the longitudinal pump (LP) indicates that it is an auspicious material for an optical diode-pumped solid-state laser.

Figure 8b shows the relationship between input and output energy in toluene. The CO showed an efficiency of 17.85% ± 1.94%, with the maximum being 19.79%. This is due to the high optical gain coefficient, large gain cross-section, and low reabsorption of the CO. Figure 8c shows the spectra narrowing and rapid increase in intensity with respect to the increase in the pump energy (for TP). At 0.5 mJ, the FWHM dropped from 40 to 7 nm and the intensity increased to higher values. A further increase in pump energy increased the output intensity, but the FWHM of ASE was maintained. The decrease in linewidth took place because the net gain was maximal near the vibronic transition peaks of the Fluorescence spectrum (FLS); hence, the spectrum displayed gain narrowing as the pump energy increased. 

**Figure 9:**
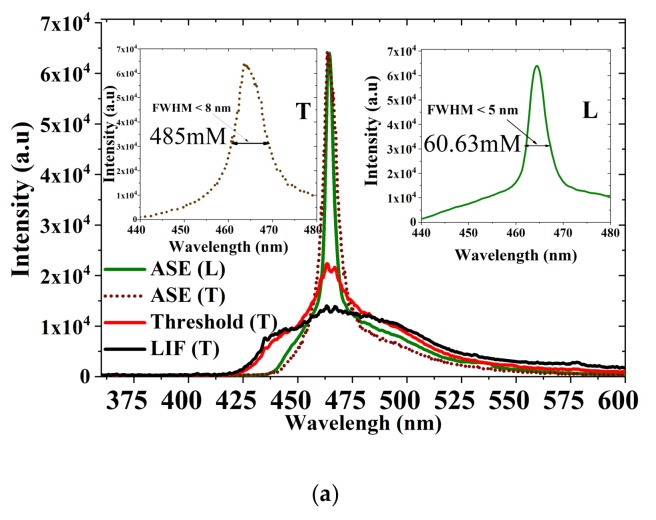

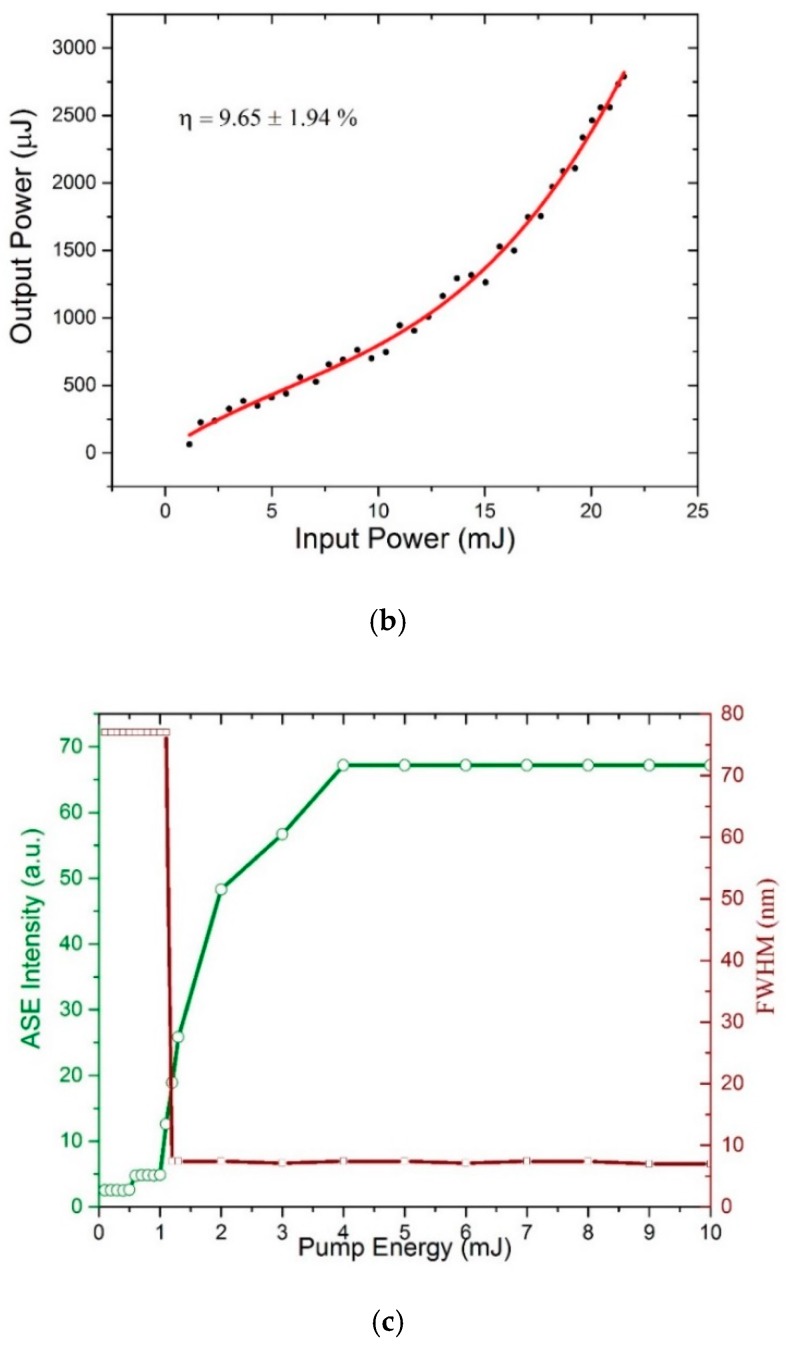
(**a**) Amplified spontaneous emission (ASE), threshold, and LIF Spectra of CO in acetone, (**b**) efficiency of CO ASE in acetone with input energy (mJ) versus output energy (μJ), and (**c**) relationship between pump energy, intensity, and FWHM of ASE in acetone.

Figure 9a shows the spectral response of the CO in acetone (a polar aprotic solvent) at the same concentration for transverse TP (485 µM) and longitudinal LP (60.63 µM), respectively. The threshold for TP was 1.2 mJ, which is twice that of the toluene environment. The FWHM was 8 nm broader (~1 nm) than the CO in toluene. Similarly, for LP, the FWHM was 5 nm and the minimum pump energy for laser action was 2.5 mJ. The ASE efficiency was 9.65% ± 1.94%, with a maximum η of 11.04% as shown in Figure 9b. The relationship between pump energy, the narrowing of FWHM, and rapid increase in ASE intensity is shown in Figure 9c, at approximately 1.5 mJ the FWHM dropped from 74 to 8 nm and the intensity increased to higher values. Due to high polarity, volatility, and plasma production the ASE in an acetone environment is rarely reported. However, the CO produces a very stable ASE output, when the solution is maintained at a low temperature (5 °C). The CO dissolved in the polar solvent through the high polarizability around the nitrogen atom of 9-ethyl-9*H*-carbazole segments, as shown in Figure S1.

### 3.7. TRS of the CO BECV-DHF in Toluene

**Figure 10:**
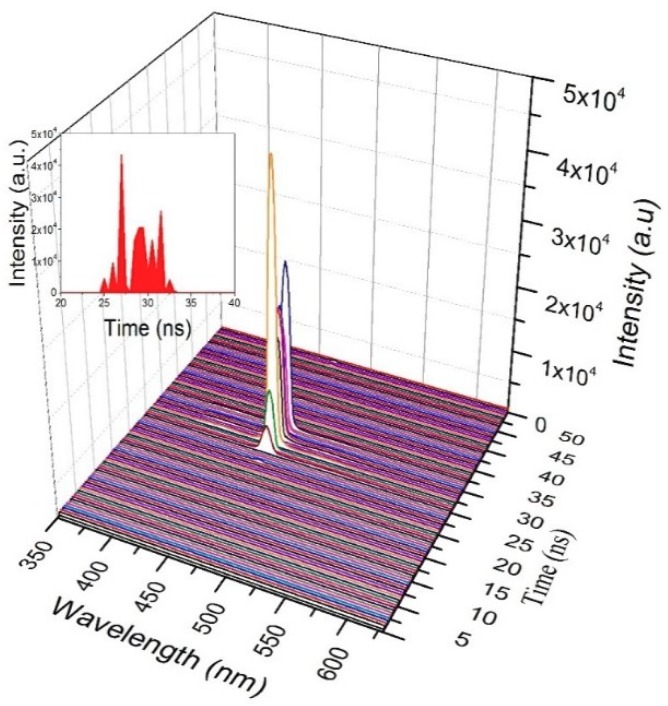
Time dynamics of CO in toluene at pump energy of 1.25 mJ and concentration of 485 µM.

Even though we studied the sub-nanosecond time dynamics of CO in various solutions, we present only the dynamics of CO in toluene solutions under TP. Figure 10 shows the TRS spectra of CO in toluene, at a low pump energy of 1.25 mJ and concentration of 485 µM; it took 25 ns from the trigger to start fluorescence. However, due to the high gain cross-section, the CO produced ASE in the next 1 ns, and the high-intensity ASE was abruptly stopped within 3 ns, due to the rapid depletion of the excited state species through stimulated emission. For the next 2 ns, a momentary blink (no ASE) occurred, during which the trailing edge of the pump pulse excited the ground state species and ASE was produced in a constant phase and completely decayed. A weak fluorescence due to the relaxation process was produced at approximately 49 ns. This dynamic shows that this CO is capable of rapid excitation and fluorescence processes. 

**Figure 11:**
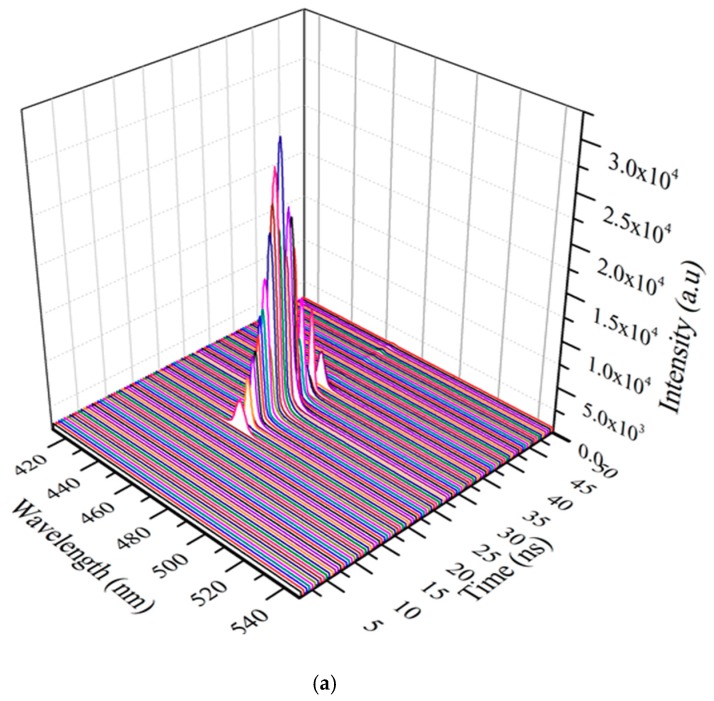

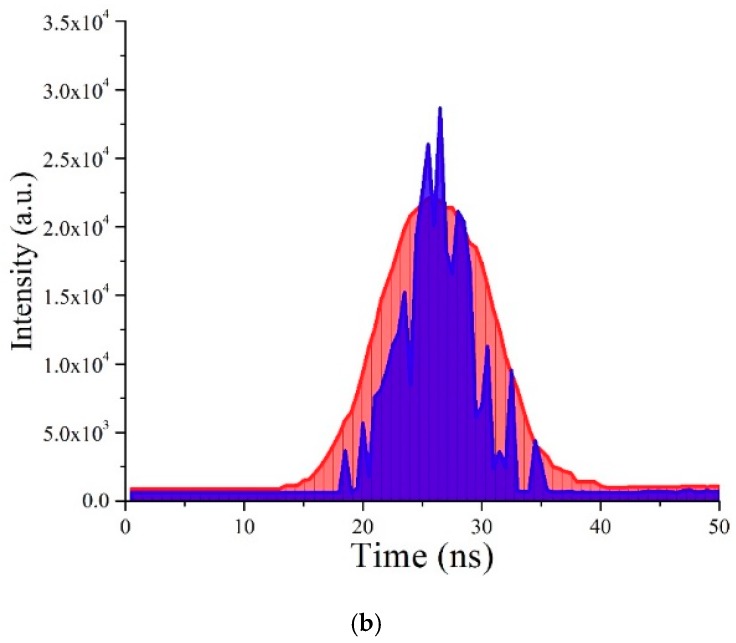
(**a**) Time dynamics of CO in toluene at pump energy of 4 mJ and concentration of 485 µM and (**b**) average temporal profile of the peak ASE wavelength.

Figure 11a shows the time-dependent response of the CO in toluene at a concentration of 485 µM for TP with pump energy of 4 mJ. The ASE produced by the CO was temporally Gaussian, with a deviation with 96% ± 1% conformation. Figure 11b shows the actual ASE output (peak intensity at 464 nm) and a smoothed average of the same (5-point adjacent average) to visualize the near-Gaussian temporal ASE profile. The CO process temporal self-beam shaping ability due to second-order nonlinear effects. The experimental results indicate that the pump beam excites the continuous and that simulated emission was also maintained at a constant rate. This phenomenon shows that this CO at this concentration in toluene forms a perfect four-state laser, which is suitable as a continuous wave laser, a property yet to be explored. 

The temporally Gaussian ASE was produced for a range of pump energy of 1.5–6 mJ. This rare phenomenon of temporal Gaussian ASE shows the high efficiency of this CO.

**Figure 12:**
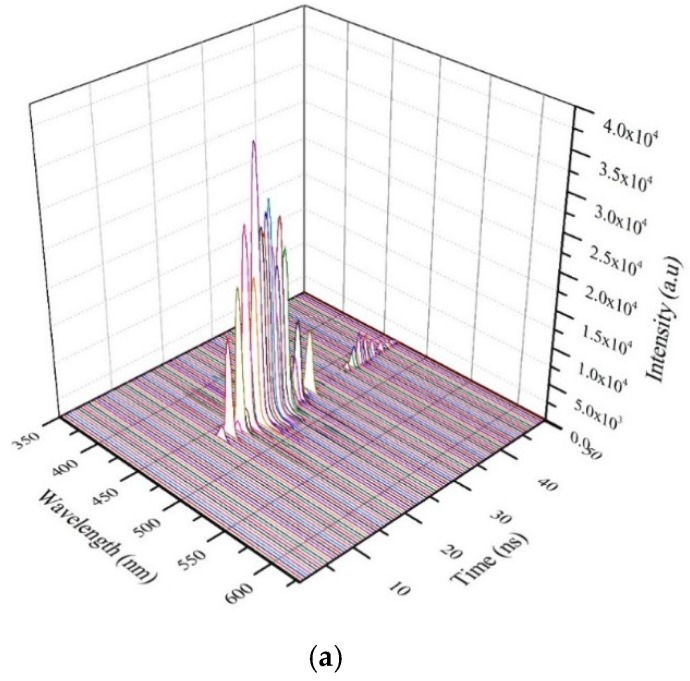

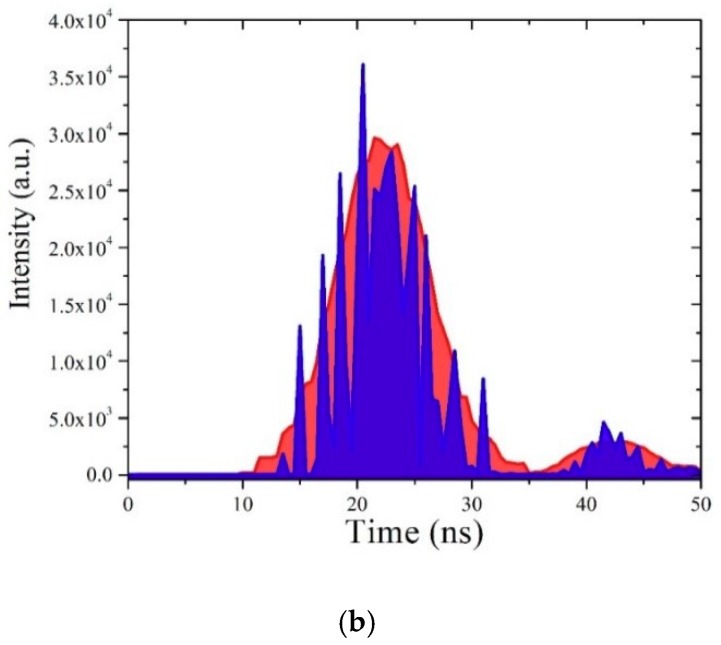
(**a**) Time dynamics of CO in toluene at pump energy of 12 mJ and concentration of 485 µM and (**b**) average temporal profile of the peak ASE wavelength at a pump energy of 12mJ.

Figure 12a shows the time dynamics of CO in toluene at the above-discussed concentration, but the pump energy was increased above 6 mJ. When the pump energy was 12 mJ, due to high photon flux, the temporal Gaussian shape of the output pulse was slightly modified, the ASE attained the peak in the rapid phase, momentarily dropped to less than half of its strength, and started again and stabilized for approximately 4 ns; finally, the primary pulse rapidly fell. However, after 5 ns, the secondary ASE appeared with a much weaker intensity and had a miniature image of the primary pulse. This is an indication of nanosecond relaxation due to high photon flux and excited state absorption [39]. The actual and average temporal profile of the peak ASE wavelength is depicted in Figure 12b. Figure 12b shows the actual ASE output (peak intensity at 464 nm) (blue shape) and smoothed average of the same (5-point adjacent average) (red shape) to visualize the near-Gaussian temporal ASE profile.

The Gaussian profile became sharp with abrupt blinks, and the peak intensity was attained faster and not in the middle. The excess energy stored in the excited state gave rise to a strong fluorescence relaxation. The profile of the relaxation was almost the same as the primary fluorescence but with only a fraction of the intensity. 

**Figure 13:**
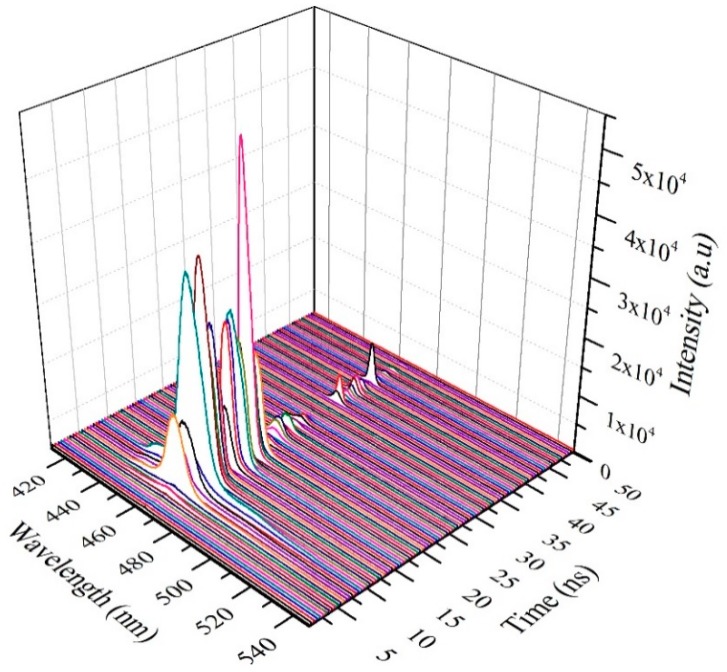
Time dynamics of CO under high pump energy (25 mJ).

Figure 13 show the consequence of high photon flux (i.e., high pump energy). In the four-level system, this oligomer can only handle pump energy up to a certain level (up to 18 mJ) in toluene. The pump energy was increased to a higher level (in this case 25 mJ), and the equilibrium between the excitation and de-excitation (radiative and nonradiative pathways) was disturbed. As a result, the smooth temporal Gaussian output of ASE was distorted. The excited-state absorption process took place and gave rise to relaxation after 4–6 ns after the primary ASE. However, this relaxation is considered to be a loss as most of the excited species relaxed through a nonradiative process.

## 4. ASE Stability

**Figure 14:**
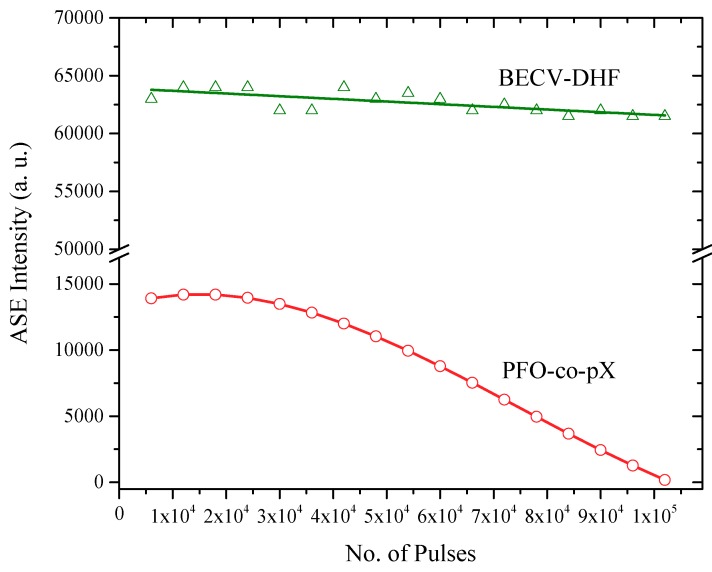
The photochemical stability of BECV-DHF and PFO-co-pX at a concentration of 250 µM.

The ASE stability of BECV-DHF (CO) and a conjugated polymer (CP) (PFO-co-pX) in toluene was measured and compared as shown in Figure 14. Both CO and CP were kept at a concentration of 250 µM, and the pump energy was 20 mJ. The output of CO was five times higher than CP. After one hundred thousand (10^5^) shots, the output of the CO was almost the same, but the macromolecule with a very similar chemical structure (at monomer level) suffered 90% damage to its performance. 

## 5. Conclusions

In this study, a high-efficient laser material was studied theoretically and experimentally with results that were in agreement with each other. The oligomer was soluble in many solvents, both in polar and nonpolar solvents due to the presence of carbazoles and the 9,9-dihexyl group, respectively. The molecule was designed to be planar, and fluorine groups contributed to the laser efficiency. The ASE was produced in most of the solvents, except methanol, ethylene glycol, and few others due to low aromaticity or aprotic polarity (for the concentration and pump energy studied in this work). The maximum efficiency was found to be 20% in toluene. The time-resolved spectra revealed the excellent temporal Gaussian ASE for the first time in oligomers. The optimal range for the oligomer to produce ASE without disturbing the equilibrium between excitation and de-excitation was 1.5–18 mJ. Any increase in pump energy produced irregular ASE amplitudes and induced strong relaxation. 

## Supplementary Materials

The following are available online at https://www.mdpi.com/2073-4360/11/10/1534/s1. Figure S1: (a) polarizability of CO BECH-DHF side view and (b) polarizability of CO BECH-DHF top view, Figure S2: The vibration of frequencies 1230–1240 cm^−1^ C=C and C-C stretching mode in rings, Figure S3: The vibration of frequencies 1230–1240 cm^−1^ C=C, C-C stretching mode in rings and C-H bending, Figure S4: The vibration of 748 cm^−1^ (T) [i.e., 742 cm^−1^ (E)] C=C and C-C stretching mode in rings, Figure S5: optical energy gap by crossing the absorption and fluorescence spectra for CO in toluene (the concentration was 0.235 µM), Figure S6: optical energy gap by crossing the absorption and fluorescence spectra for CO in acetone (the concentration was 0.235 µM), Figure S7: Absorption and fluorescence spectra of BECV-DHF in Acetone for different concentrations.
