# Supplementary material for: Optically Pumped Intensive Light Amplification from a Blue Oligomer
Source: Polymers (Basel). 2019 Sep 20;11(10):1534. doi: 10.3390/polym11101534 (PMC6835369; doi:10.3390/polym11101534)
Supplement: Supplementary file 1 [file polymers-11-01534-s001.pdf]

polymers

MDPI

Supplementary Material for:

Optically pumped Intensive light amplification from a blue oligomer .

Mamduh J. Aljaafreh[1], Saradh Prasad[1,2], Mohamad S. AlSalhi[1,2,*], Zeyad A. Alahmed[1] and Muneerah M. Al-Mogren[3]

[1] Department of Physics and Astronomy, College of Science, King Saud University, 11451 Riyadh, Saudi Arabia; maljaafreh@ ksu.edu.sa; zalahmed@ ksu.edu.sa.

[2] Research Chair on laser diagnosis of cancers, Department of Physics and Astronomy, College of Science, King Saud University, 11451 Riyadh, Saudi Arabia; srajendra@ksu.edu.sa.

[3] Chemistry Department, Faculty of Science, King Saud University, PO Box 2455, Riyadh, 11451, Saudi Arabia; mmogren@ksu.edu.sa

* Correspondence:Correspondence: malsalhi@ksu.edu.sa; Tel.: +966-50-510-4815.

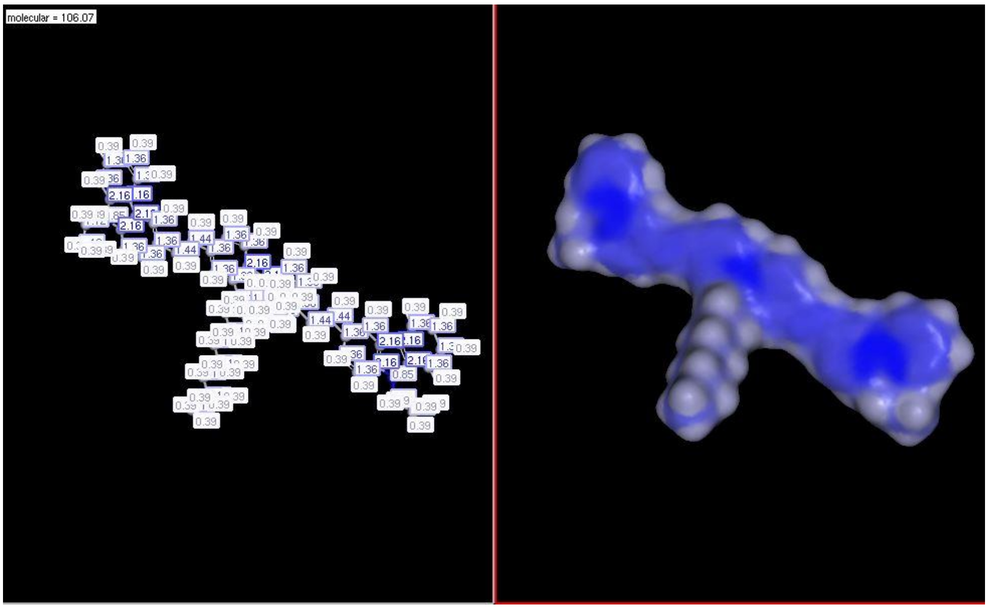

Fig. S1 (a) polarizability of CO BECV-DHF side view.

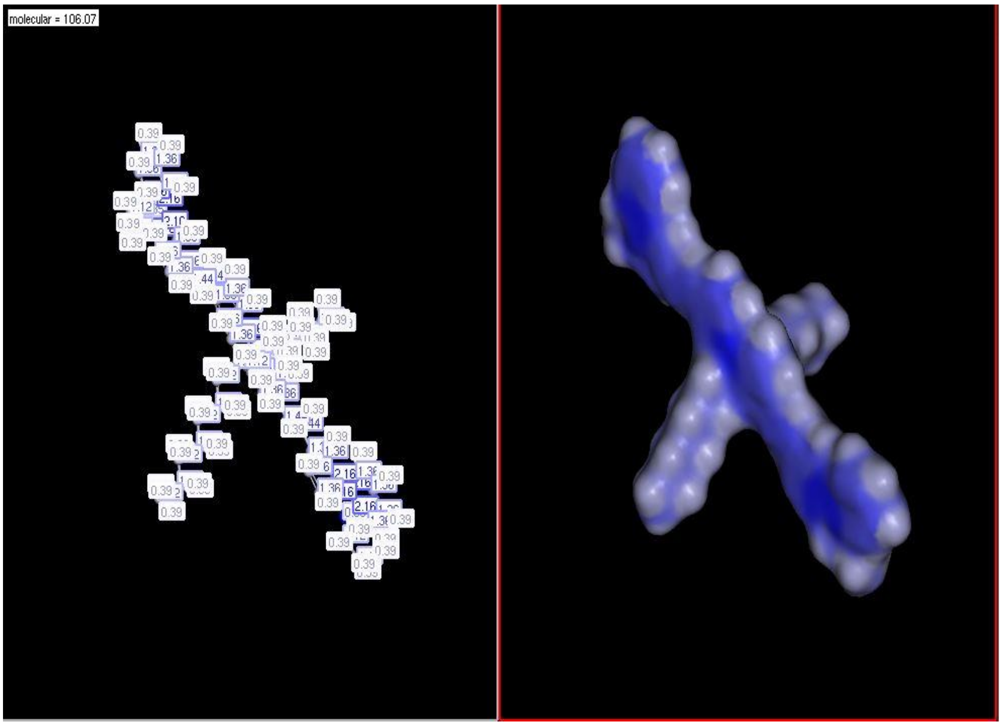

Fig. S1 (b) polarizability of CO BECV-DHF top view.

polymers

MDPI

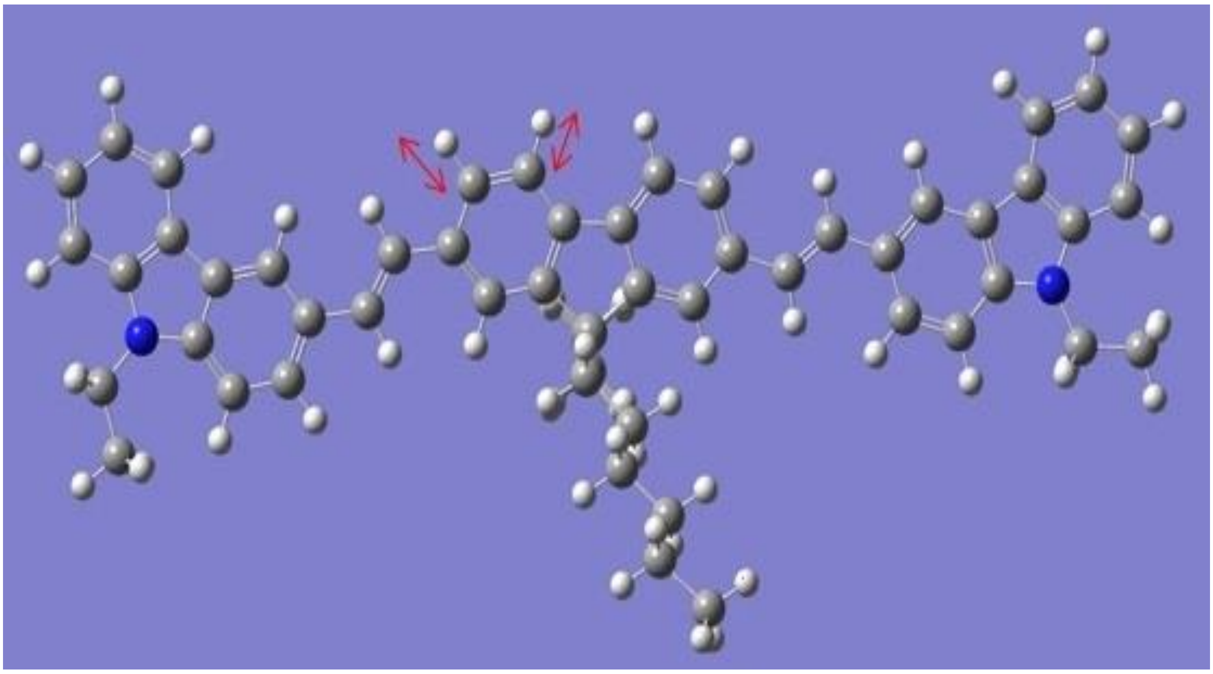

Fig. S2 The vibration of frequencies 1230-1240 cm^{-1} C=C and C-C stretching mode in rings

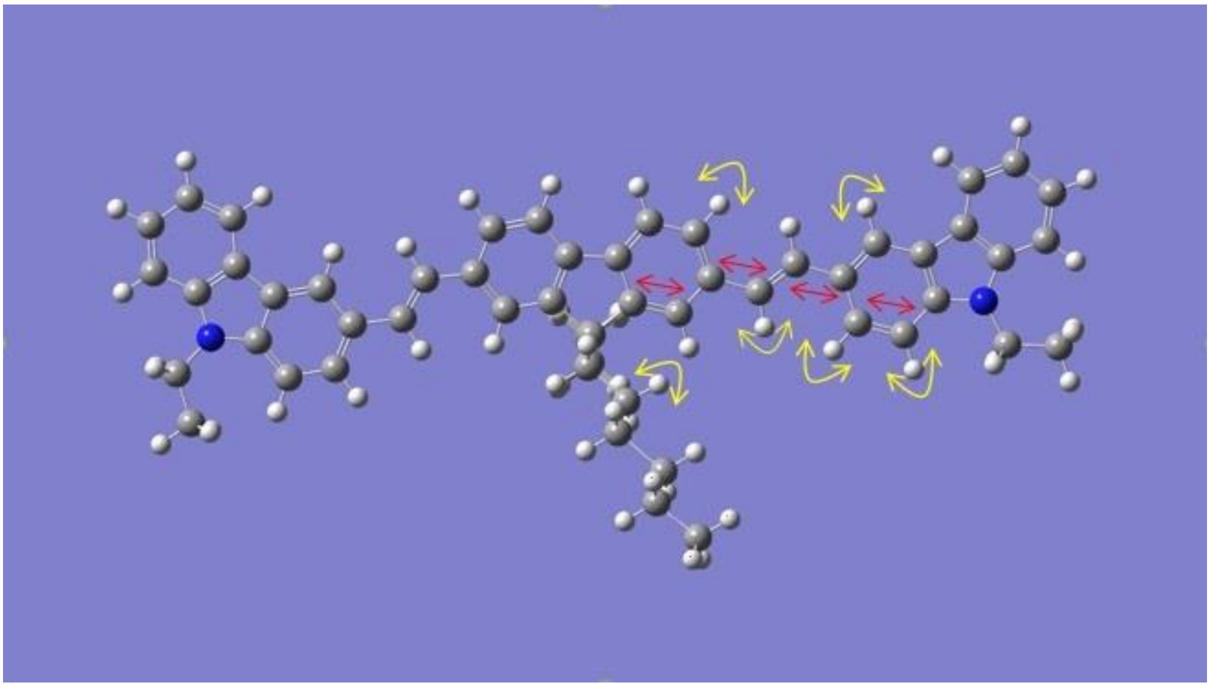

Fig. S3 The vibration of frequencies 1230-1240 cm^{-1} C=C, C-C stretching mode in rings and C-H bending

polymers

MDPI

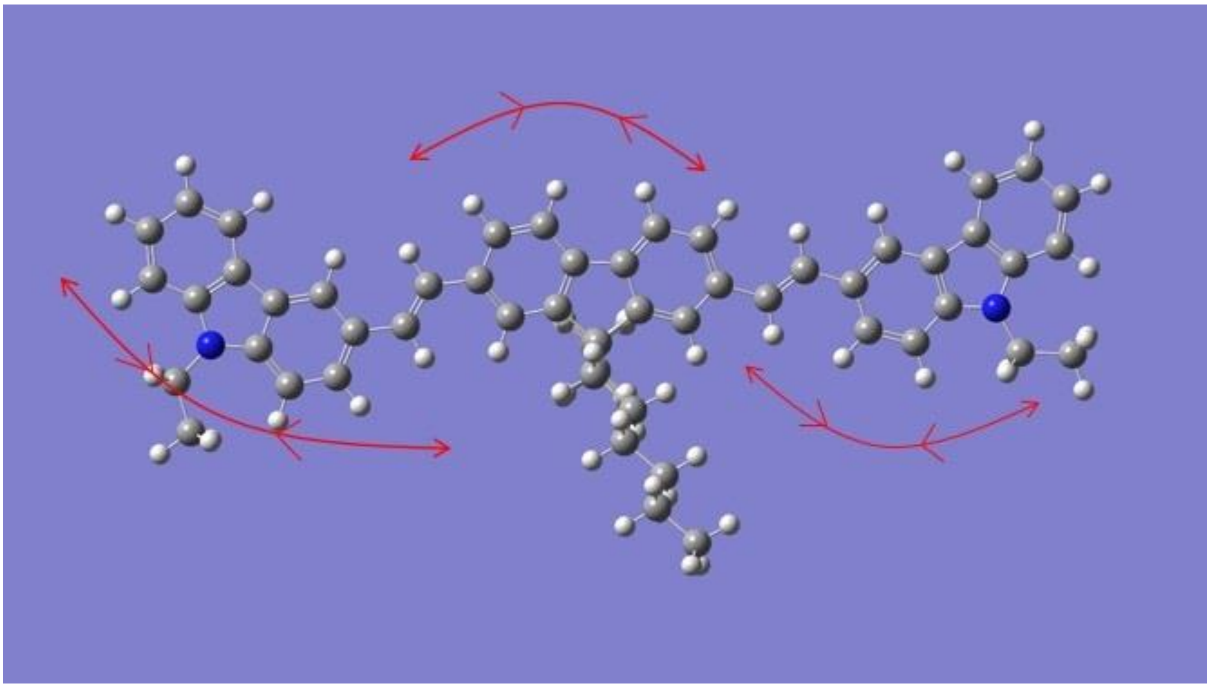

Fig. S4 The vibration of 748 cm[-1] (T) [i.e 742 cm [-1] (E)] C=C and C-C stretching mode in rings

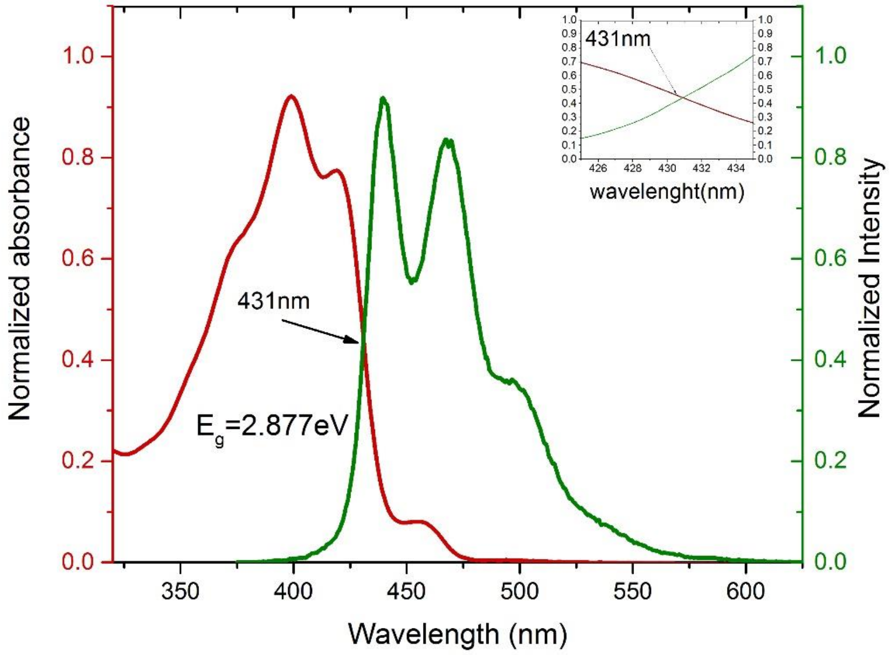

Fig. S5 optical energy gap by crossing the absorption and fluorescence spectra for CO in toluene (the concentration was 1.235μM)

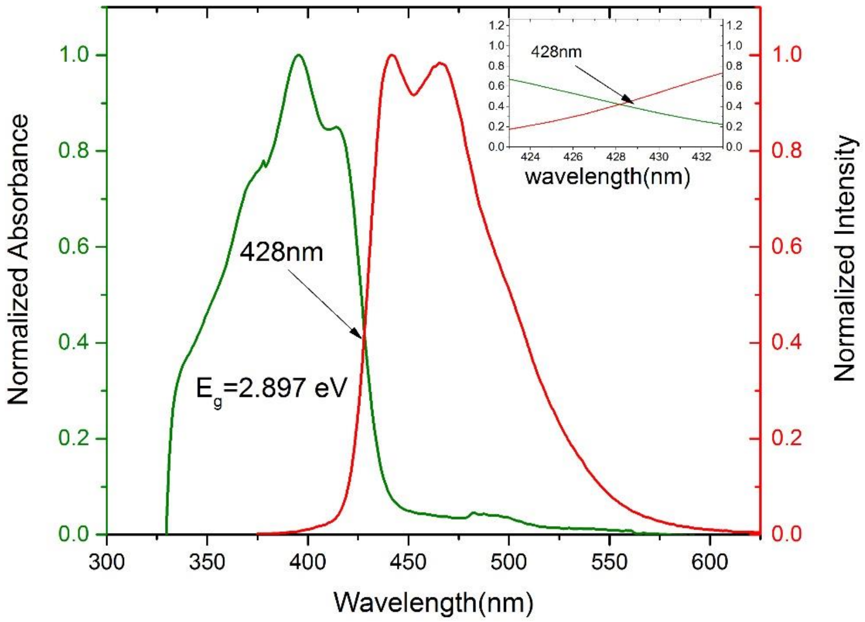

Fig. S6 optical energy gap by crossing the absorption and fluorescence spectra for CO in acetone (the concentration was 1.235 μM)

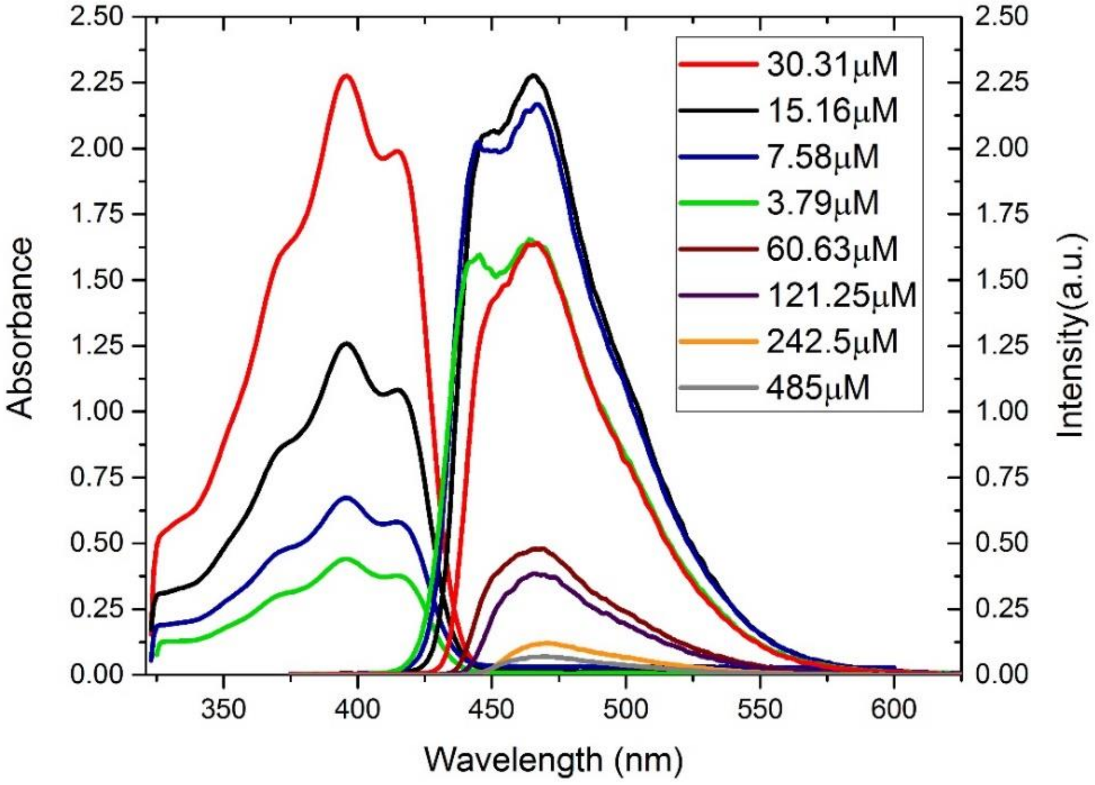

Fig. S7 Absorption and fluorescence spectra of BECV-DHF in Acetone for different concentrations.
